# psygenet2r: a R/Bioconductor package for the analysis of psychiatric disease genes

**DOI:** 10.1093/bioinformatics/btx506

**Published:** 2017-08-17

**Authors:** Alba Gutiérrez-Sacristán, Carles Hernández-Ferrer, Juan R González, Laura I Furlong

**Affiliations:** 1 Research Programme on Biomedical Informatics (GRIB), Hospital del Mar Medical Research Institute (IMIM), Barcelona, Spain; 2 Department of Experimental and Health Sciences (DCEXS), Universitat Pompeu Fabra (UPF), Barcelona, Spain; 3 ISGlobal, Centre for Research in Environmental Epidemiology (CREAL), Barcelona, Spain; 4 CIBER Epidemiología y Salud Pública (CIBERESP), Barcelona, Spain

## Abstract

**Motivation:**

Psychiatric disorders have a great impact on morbidity and mortality. Genotype–phenotype resources for psychiatric diseases are key to enable the translation of research findings to a better care of patients. PsyGeNET is a knowledge resource on psychiatric diseases and their genes, developed by text mining and curated by domain experts.

**Results:**

We present psygenet2r, an R package that contains a variety of functions for leveraging PsyGeNET database and facilitating its analysis and interpretation. The package offers different types of queries to the database along with variety of analysis and visualization tools, including the study of the anatomical structures in which the genes are expressed and gaining insight of gene‘s molecular function. Psygenet2r is especially suited for network medicine analysis of psychiatric disorders.

**Availability and implementation:**

The package is implemented in R and is available under MIT license from Bioconductor (http://bioconductor.org/packages/release/bioc/html/psygenet2r.html).

**Supplementary information:**

Supplementary data are available at *Bioinformatics* online.

## 1 Introduction

Research in the genetics of psychiatric diseases generates a large amount of data dispersed in different repositories or only available in free text as publications. Knowledge platforms offering genotype–phenotype information for psychiatric diseases are required to support translational research in psychiatry. PsyGeNET (Gutiérrez-Sacristán *et al.*, 2015, 2017) is a knowledge resource that collects and validates information of psychiatric diseases and their genes. The current release of PsyGeNET contains updated information on depression, alcohol and cocaine use disorders, and has been expanded to cover other psychiatric diseases, such as bipolar disorder and schizophrenia (Gutiérrez-Sacristán *et al.*, 2017). The PsyGeNET database is developed from information extracted from the literature by text mining tools (Bravo *et al.*, 2015), followed validated by a team of experts (Gutiérrez-Sacristán *et al.*, 2017). The information in PsyGeNET is standardized using community-driven standards, and the evidence supporting the association of a gene to a disease is recorded and available to the user, including contradictory or conflictive findings. By using PsyGeNET the user can gain insight on the molecular basis of psychiatric disorders and their comorbidities. The psygenet2r package has been developed to facilitate the query and analysis of PsyGeNET data and to allow its integration with other packages available in R to develop bioinformatic analysis workflows. Another advantage of using the psygenet2r package compared to the web interface is the variety of data visualization formats provided, such as networks, heatmaps and barplots. Moreover, psygenet2r enables the user to: (i) retrieve the genes associated to PsyGeNET diseases, (ii) annotate a user‘s list of genes with PsyGeNET diseases, (iii) explore the molecular functions of the proteins encoded by the genes, (iv) analyse the tissues/anatomical structures in which the genes are expressed, (v) explore data provenance, such as number of publication and the Evidence Index and (vi) analyze the similarity between diseases based on shared genes.

## 2 The psygenet2r package

### 2.1 Data input

psygenet2r package allows retrieving and exploring PsyGeNET information using a specific gene or disease, or a list of them, using a variety of identifiers. The ‘score’ argument is based on the PsyGeNET Evidence Index (EI), which quantifies the level of supporting evidence for a gene-disease association (GDA). For instance, an EI of 1 indicates that all the evidence collected in PsyGeNET supports the association of the gene with the disease, while an EI of 0.5 indicates that there are contradictory findings for a particular GDA. 

### 2.2 S4 objects defined in psygenet2r

**Fig. 1:**
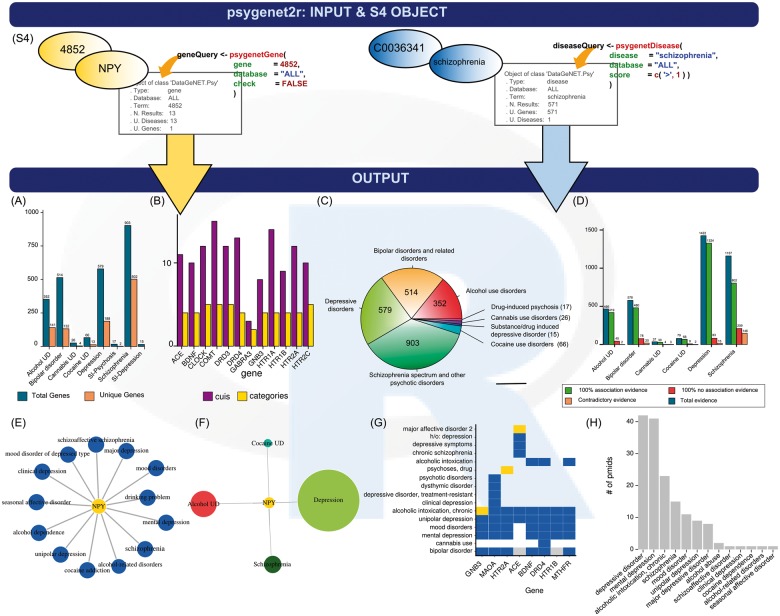
The psygenet2r package: (S4) Input data type DataGeNET.Psy object structure. Psygenet2r allows free text strings or standard identifiers for both genes and diseases. Different analysis and their visualization option: (**A**) bar-plot showing the genes associated with each disease category; (**B**) bar-plot showing the disease concepts and disease categories associated with each gene; (**C**) pie chart showing the number of genes per disease category; (**D**) bar-plot showing the type of association; (**E**) gene-disease association network; (**F**) gene-disease category association network; (**G**) evidence index heatmap and (**H**) barplot showing the number of publications supporting gene-disease association

To provide easy data storage and manipulation, we created two S4 class objects, DataGeNET.Psy and JaccardIndex.Psy. The DataGeNET.Psy object is obtained when using the query functions psygenetGene and psygenetDisease, and is used as input for the rest of psygenet2r functions, such as the plot function. The DataGeNET.Psy object (Fig. 1) contains a summary of the search and the results obtained. The JaccardIndex.Psy object is obtained when the jaccardEstimation function is applied, and computes the Jaccard Index (JI) as an estimation of the similarity of two diseases based on shared genes. JaccardIndex.Psy object contains a summary of the search, the parameters used for the estimation and the results.

### 2.3 Visualization of GDAs

psygenet2r makes a special focus on visualizing the results, providing a variety of representation formats, such as networks, heatmaps and bar-plots. The results can be visualized in different ways by applying the plot and geneAttrPlot functions. Depending on the type of object used, the function and the type argument, the user can explore the different GDA attributes (Supplementary Table S1). Visualizing the results according to the different attributes, such as the EI, the number of publications or the source database, is a functionality provided by the psygenet2r package that is not available in PsyGeNET web.

### 2.4 Enrichment analysis

Two different psygenet2r functions—enrichedPD and topAnatEnrichment—can be used to characterize the list of genes. The function enrichedPD performs an enrichment analysis using the diseases from PsyGeNET, while the topAnatEnrichment function is based on the expression of genes in anatomical structures from Bgee database (Bastian *et al.*, 2008; Komljenovic *et al.*, 2016). The result for both functions is a data frame containing the enrichment results with the associated p-value.

### 2.5 Assessing the similarity among diseases based on shared genes

With psygenet2r, we can assess the similarity between two diseases based on the number of shared genes. The disease similarity is obtained using the Jaccard Index (JI), and the significance of the JI obtained is estimated by a bootstrap procedure implemented in the Jaccard estimation function from random disease gene sets obtained from DisGeNET (Piñero *et al.*, 2017). The results of this analysis can be visualized using a bar-plot or a heatmap (Supplementary Table S1).

## 3 Case study

An example of the application of psygenet2r can be found in the analysis of genes identified in a GWAs study on bipolar disorder, showing how it can be used to analyse a set of genes provided by the user (Supplementary File S2). A detailed description on psygenet2r functions can be found in the vignette (Supplementary File S1).

## 4 Conclusion

psygenet2r is an R package for gaining insight on the molecular basis of psychiatric disorders and their comorbidities. psygenet2r imports data from PsyGeNET database, a knowledge resource on psychiatric disorders and their genes, and can be integrated with other R packages. psygenet2r also implements several functions to visualize and analyse the results in a clear and meaningful way. psygenet2r is especially suited for network medicine analysis of psychiatric disorders.

## Funding

This work was supported by ISCIII-FEDER [PI13/00082, CP10/00524, CPII16/00026], MICINN [MTM2015-68140-R], IMI-JU under grants agreements no. 115191 (Open PHACTS), no. 115372 (EMIF), no. 115735 (iPiE), resources of which are composed of financial contribution from the EU-FP7 [FP7/2007–2013] and EFPIA companies in kind contribution, and the EU H2020 Programme 2014–2020 under grant agreements no. 634143 (MedBioinformatics) and no. 676559 (Elixir-Excelerate). The Research Programme on Biomedical Informatics (GRIB) is a member of ELIXIR-ES and the Spanish National Bioinformatics Institute (INB), PRB2-ISCIII and is supported by grant PT13/0001/0023, of the PE I + D+i 2013-2016, funded by ISCIII and FEDER. A.G.S. acknowledges financial support from the Spanish Ministry of Economy and Competitiveness, through the ‘María de Maeztu’ Programme for Units of Excellence in R&D [MDM-2014-0370].

*Conflict of Interest*: none declared.

## Supplementary Material

Supplementary Data
