# Supplementary material for: psygenet2r: a R/Bioconductor package for the analysis of psychiatric disease genes
Source: Bioinformatics. 2017 Aug 17;33(24):4004–6. doi: 10.1093/bioinformatics/btx506 (PMC5860088; doi:10.1093/bioinformatics/btx506)
Supplement: Supplementary Data [file btx506_psygenet2rv8.0_supplementary.docx]

Supplementary information of the manuscript “*psygenet2r: a R/Bioconductor package for the analysis of psychiatric disease genes*”.

Authors: Alba Gutiérrez-Sacristán, Carles Hernández-Ferrer, Juan R. González, Laura I. Furlong

File S1: R package vignette (file name: psygenet2rVignette.pdf, available at https://bioconductor.org/packages/release/bioc/vignettes/psygenet2r/inst/doc/general_overview.html)

File S2: Case study on a GWAs of bipolar disorder (file name: psygenet2rCaseStudy.pdf, available at https://bioconductor.org/packages/release/bioc/vignettes/psygenet2r/inst/doc/case_study.html)

Table S1. Visualization options. DC = Disease Category; **GDCAs = Gene Disease Class Associations

| Input object | psygenet2r function | Argument type | Output |
| --- | --- | --- | --- |
| DataGeNET.Psy | geneAttrPlot | disease category | genes associated with each DC* (Fig1.A) |
|  |  | gene | disease concepts and DC associated with each gene (Fig1.B) |
|  |  | pie | Pie chart nº of genes per DC (Fig1.C) |
|  |  | evidence index | Bar-plot showing the type of association (Fig1.D) |
|  | plot | GDA network | GDA network (default type) (Fig1.E) |
|  |  | GDCA network | GDCAs** network (Fig1.F) |
|  |  | GDA heatmap | Evidence Index heatmap (Fig1.G) |
|  |  | GDCA heatmap | GDCAs heatmap (gene); EI heatmap for GDA (disease) |
|  |  | publications | nº of publications supporting GDA (Fig1.H) |
| JaccardIndex.Psy | plot | None | JI Bar-plot/heatmap |
| Gene vector | pantherGraphic | None | Barplot showing the protein panther class |
